# Biomonitoring of Acetylcholinesterase (AChE) Activity among Smallholder Horticultural Farmers Occupationally Exposed to Mixtures of Pesticides in Tanzania

**DOI:** 10.1155/2019/3084501

**Published:** 2019-09-11

**Authors:** Jones A. Kapeleka, Elingarami Sauli, Omowunmi Sadik, Patrick A. Ndakidemi

**Affiliations:** ^1^The Nelson Mandela African Institution of Science and Technology (NM-AIST), P.O. Box 447, Arusha, Tanzania; ^2^Tropical Pesticides Research Institute (TPRI), P.O. Box 3024, Arusha, Tanzania; ^3^Binghamton University-State University of New York, Binghamton, NY 13902, USA

## Abstract

Biomonitoring of pesticides exposure has currently become a matter of great public concern due to the potential health effects of pesticides. This study assessed levels of acetylcholinesterase (AChE) inhibition and associated health effects in uncontrolled smallholder farming systems in rural Tanzania. A cross-sectional study was conducted of 90 exposed farmers and 61 nonexposed controls from horticultural zones. A structured questionnaire was administered, and a capillary blood sample of 10 *μ*l was used to measure AChE activity using an Erythrocyte Acetylcholinesterase Test Mate Photometric Analyzer kit (Model 400). A multiple logistic regression model was used to investigate determinants of pesticide exposure. The study revealed that smallholder farmers are occupationally exposed to pesticides. Exposed farmers had significantly lower AChE levels. The use of personal protective equipment (PPE) did not significantly reduce the likelihood of AChE inhibition. Women, younger and older farmers, and underweight, overweight, and obese farmers were at increased risk of AChE inhibition. Increase in age (10 years) increased likelihood of AChE inhibition by 6.7%, while decrease in BMI increased likelihood of AChE inhibition by 86.7% while increased pesticides contact hours increased risk of having lower AChE at about 3 times. The number of exposure symptoms (14.10 ± 7.70) was higher in exposed farmers than unexposed. Self-reported symptoms are confirmed to correlate to lower AChE. Prevalence of tiredness (71.6% against 15.5%), fatigue (64.8% against 27.6%), soreness in joints (59.1% against 20.7%), thirst (52.3% against 12.1%), skin irritation (52.1% against 17.2%), salivation and abdominal pain (50% against 8.6% and 31.0%, respectively), muscle weakness (47.7% against 24.1%), and memory loss (47.7% against and 29.3%) differed significantly between exposed and control. This study provides useful information regarding the level of occupational and environmental exposure to pesticides in smallholder horticultural production systems. Pesticides use needs to be controlled at farm level by developing pesticides monitoring and surveillance systems.

## 1. Introduction

The use of pesticides in controlling crop pests and boost agriculture production had increased in the recent past due to their rapid knockdown effect [1]. Smallholder horticultural production systems constitute the main subsector where pesticides are highly used. The uncontrolled nature, poor usage of personal protection equipment and lack of adequate knowledge about pesticide use results in serious health consequences [2].

Organophosphates (OPs) and carbamates (CA) are the most used families of pesticides [3–5]. These chemical compounds are antiacetylcholinesterase and endocrine-disrupting substances. OPs and CA exert their toxicity by interfering with the normal function of acetylcholine hydrolysis, a necessary task for synaptic response and an essential neurotransmitter in the autonomic and central nervous system [6, 7]. High exposure to these chemicals results in neurotoxicity as well as decreased acetylcholinesterase (AChE) activity [8–10]. Hormonal changes, abnormal sperm, ovaries and eggs production, neurological, gastrointestinal, dermatological, and respiratory manifestations among many other effects are associated with acetylcholine inhibition due to organophosphorus pesticides exposure [11, 12].

People who have direct and prolonged contact with pesticides have more risk of exposure [13]. Increased risk of exposure is a result of pesticides accumulation on the clothing, skin, and boots after spraying in the field as well as inhalation exposures through spray residue and spray drift [14]. Spraying pesticides in mixtures as opposed to recommended spraying practice on the pesticides labels foster occupational exposure [1, 15].

In evaluating potential human exposure to chemicals that act as cholinesterase inhibitors, erythrocyte blood cholinesterase (AChE) testing is done to establish the levels of exposure [10, 16, 17]. AChE inhibition provides a useful biomarker of pesticides exposure and toxicity in human body and is more indicative of the severity of pesticides poisoning [18, 19]. But owing to the fact that interindividual variability in AChE biomonitoring is much higher than intraindividual variability in ChE among the normal population [20], a single measure taken to establish the levels of severity of exposure may overestimate or underestimate the actual exposure.

Currently, few AChE studies from Tanzania had been conducted in traditional cash crops, including coffee, commercialized tea estates, and flower industries targeting exposed farmers only [21, 22]. In one study, no significant difference in the levels of AChE among 133 coffee and cotton smallholder farmers between farming (spraying) and nonfarming (nonspraying) season was reported [21]. Furthermore, , farmers in commercialized tea estates and flower industries were occupationally exposed to pesticides, but unexposed control groups were not included in both cases. The most recent study conducted in Arusha assessed the health symptoms of pesticides exposure among flower and onion pesticides applicators indicated neurological health symptoms and AChE inhibition [23]. The available studies assessed exposed farmers only and household survey data to examine the health effects of pesticide exposure; hence, the need to use objective methods to evaluate health effects of pesticide exposure using unexposed individuals as control group.

Due to the differences in levels of exposure, type of pesticides used, regulatory mechanisms, pesticides mixture compositions, geographical behaviors, and demographic characteristics of the farming population areas, it is difficult to generalize and compare the published studies [24, 25]. The aim of this study was, therefore, to assess the comparative levels of pesticides exposure by determining the AChE levels and factors influencing pesticides exposure in smallholder horticultural in Tanzania, to describe pesticides-handling practices and risk behaviors and to derive an association between AChE inhibition and self-reported symptoms. The study compares levels of exposure and self-reported symptoms with AChE levels between farmers and control group, complementing previous studies where unexposed control groups were not included.

## 2. Materials and Methods

### 2.1. Study Design

This was a comparative cross-sectional study conducted among 90 farmers occupationally exposed to pesticides and 61 healthy individuals (control group) with similar socioeconomic characteristics. Participants were selected from the general population for controlling the effects of confounding factors including lifestyles, exposure to X-rays, eating habits, inter and intraindividual variations, weather, and geographical characteristics on AChE inhibition. However, the study tried to minimize these limitations by purposive selection of the control group with matching demographic characteristics and geographical locations as well as adjusting for age and BMI in the multivariate analysis. This comparative use of control groups in drawing conclusive evidence of pesticides exposure had been reported in several studies [8, 10, 26–30].

### 2.2. Sample Size and Sampling Procedure

Kilolo (Iringa) and Ngarenanyuki (Arusha) were purposely selected based on horticultural productivity and extensive use of synthetic pesticides. Horticultural farmers were randomly selected from the list of households provided by respective village government officers. The sample was chosen based on the proportion of farmers involved in smallholder horticultural production. According to the Tanzania 2012 Population and Housing Census, Kilolo recorded 218,130 while Ngarenanyuki 20,379 farmers.

Inclusion criteria included individuals who are occupationally involved in pesticides handling and working in a sprayed field, had sprayed during the last week before survey, or had weeded/harvest field sprayed with pesticides during the same period. Exclusion criteria included individuals who are not involved directly in handling pesticides or not working in pesticides-related activities. The control group was purposely selected based on the criteria that none of them had been recently exposed to agrochemicals or previous occupational exposure to pesticides and matched with age, sex, and other demographic variables. This group included office employees and shopkeepers living in the same region as exposed individuals.

The sample size used in this study was based on previous studies which indicated that a minimum sample size of 30 farmers would be sufficient to detect a difference in cholinesterase activity between farming and nonfarming groups and that of 90 yields power over 80% [10, 17]. A total of 90 exposed and 90 unexposed individuals were recruited. From the unexposed group, 29 individuals did not meet the inclusion criteria and, therefore, were removed from the sample. Hence, a sample size of 61 unexposed individuals was used.

### 2.3. Data Collection

A structured questionnaire containing both closed- and open-ended questions was administered to participants for a health survey. The questionnaire used in the previous study [21] was used with minor modifications to suit the current research. This improved questionnaire was further pretested among 20 individuals from one village in the study areas, which was finally removed from the sample. Collected information included pesticides used, pesticides use practices and handling, frequency of application, areas sprayed, use of PPE, and exposure risk behaviors. Demographic information and farmer habits and lifestyles (age, gender, alcohol consumption, and smoking) were also collected. Assessment of exposure symptoms to OPs and CA was done through a random list of 38 different symptoms typical to OPs and CA exposure. Anthropometric measurement (height and weight) were also taken to determine the body mass index (BMI), which was calculated and categorized using four WHO criteria for underweight, normal, overweight, and obese. Field observations were done to observe pesticides mixing, handling, type of PPE used, and disposal methods of empty pesticides containers.

## 3. Determination of Acetylcholinesterase (AChE) Inhibition

### 3.1. Blood Samples Collection and Handling

Collection of blood was carried out according to the procedures explained by Cotton et al. [17] and Neupane et al. [10]. The Erythrocyte Acetyl cholinesterase Test Mate Photometric Analyzer kit (Model 400) was used to test the cholinesterase inhibition based on manufactures' standard methodology [31], whereby a capillary blood sample of 10 *μ*l was used to measure AChE activity standardized against whole blood haemoglobin. This haemoglobin-adjusted erythrocyte acetylcholinesterase activity (*Q*) was measured in (U/g Hb) and used to describe the levels of exposure.

### 3.2. Data Analysis

Statistical analysis of data was done using SPSS 22.0 computer software. Descriptive statistics such as frequencies, percentages, mean, and standard deviations were performed to summarize the characteristics for the study population, and results are presented as (mean ± standard deviation). Association of risk behaviors including smoking, eating, and use of PPE, BMI, and haemoglobin-adjusted erythrocyte acetylcholinesterase activity (*Q*) was done using chi-squared testing. One-way ANOVA testing was used to determine factors influencing haemoglobin-adjusted erythrocyte acetylcholinesterase activity (*Q*) among exposed and control subjects, which was dichotomized into two categories. The first category is “inhibition” (24.5–31.3 U/g Hb), and the second is “severe inhibition” (<24.5 U/g Hb). The cutoff point of exposure set at 24.5 U/g Hb was used. This is the recommended guidelines from the Tropical Pesticides Research Institute (TPRI), which is the regulatory threshold level indicating exposure (TPRI, 2000). Student's *t*-tests were used to compare the significant difference in the levels of exposure between farmers and control groups while the chi-square test was used to test association between the symptoms and AChE inhibition.

Multiple logistic regression analysis with the outcome variable, the probability of having low AChE level, was used to determine the critical explanatory factors for AChE inhibition. Significant level for the results was accepted at *p* < 0.05. The following logistic regression model with dummy variables to control for any individual differences was developed:

(1) $$\begin{array}{c} \text{pesticides exposure }=\;f\left(\text{age, BMI, WEP, AAS, CHP, SBP, }\varepsilon \right), \end{array}$$

where pesticides exposure is the measure of AChE inhibition indicated by low or high *Q* level, as dichotomized at one SD below the mean *Q*, i.e., at the 25.2 U/g Hb. For the explanatory variables, age is the age category of farmers, BMI is the WHO body mass index categories, WEP is the working experience with pesticides, and AAS is the average area sprayed per day in acres. Furthermore, CHP is the contact (working) hours with pesticides, SBP is the spraying break period before embarking on another intensive spraying season, and *ε* denote the unknown variables.

### 3.3. Ethical Clearance

Ethical clearance was obtained from Tanzania's National Institute of Medical Research (NIMR) with Reference No. NIRM/HQ/R.8a/Vol.IX/2742. Both farmers and unexposed individuals each signed a written consent form for a blood test and participation in the research.

## 4. Results

The study involved 108 participants from Iringa (71.5%) and 43 from Arusha (28.5%) regions in Tanzania, where smallholder horticultural production is highly practiced. In drawing comparative results from exposure, 90 exposed farmers (59.6%) and 61 control groups (40.4%) were involved in the study. The farming population was found to be generally younger. The dominant age group was between 30 and 39 years, while the mean age was (38.74 ± 12.72) years (mean ± SD), dominated by men (73.5%) compared with women (26.5%). Just above half (53.6%) had been working with pesticides for over ten years and (27.5%) for 5–9 years, as presented in Table 1, showing the demographic characteristics of the study population.

Organophosphorus (97.6%) and carbamate (54.1%) pesticides constitute the main chemical families of pesticides used in horticultural production consisting mainly of insecticides (69.0%) and fungicides (30.1%). Others include substituted benzene, pyrethroids, and organochlorines (Table 2). Furthermore, 85.5% of all registered pesticides used to fall under Class II (Moderately hazardous) WHO hazard classification of pesticides, while the rest falls under WHO Class U (unlikely to present acute hazard in regular use).

The use of personal protective equipment (PPE) was not common among farmers. None of the farmers had complete body protection, and most (85.7%) were partially protected, while 14.3% were completely unprotected. Gumboots were the major PPE widely used by farmers (83.3%). Nonetheless, farmers in onion production did not wear gumboots completely to avoid the destruction of onion bulbs. Moreover, gloves (92.9%), respirators (95.2%), masks (90.5%), goggles (97.6%), and overalls/overcoats (92.3%) were not used during pesticides handling (Table 3). The *t*-test showed no statistical difference in the AChE inhibition between farmers without protection and those who partially covered themselves during spraying (*p*=0.711).

Results from the AChE tests show that exposed farmers had a decreased AChE activity (28.05 ± 3.88 U/g Hb) compared with the control group (32.87 ± 4.36 U/g Hb). The AChE inhibition of exposed farmers (67.8%) recorded 24.5–31.3 U/g Hb compared with the control group (39.3%). Furthermore, 15.6% of exposed farmers had AChE levels less than 24.5 U/g Hb compared with none in the control group (Table 4). The difference in the levels of exposure was statistically significant (*p* < 0.001) between exposed and control group, signifying occupational exposure to pesticides. AChE levels in women mainly involved in weeding and harvesting of horticultural crops were lower compared with exposed men and their counterpart women from the control group (Table 5).

Body mass index (BMI) influenced farmers' exposure to pesticides. Exposed farmers with low BMI had significantly lower AChE levels. Half (50%) of exposed farmers under WHO underweight category (BMI < 18.5 kg/m^2^) recorded AChE < 24.5 U/g Hb compared with only 7.9% of exposed but with normal BMI (18.50–24.99 kg/m^2^). Chi-square tests showed a statistically significant association between exposure levels measured by *Q* and BMI within the exposed group (*p*=0.004). Furthermore, AChE inhibition showed a statistically significant trend that varied with BMI categories (*p* < 0.001). AChE activity was much depressed for underweight (26.73 ± 5.56 U/g Hb), overweight (27.32 ± 4.95 U/g Hb), and obese (21.90 U/g Hb) as opposed to normal BMI-exposed individuals (28.37 ± 3.32 U/g Hb), as presented in Table 6.

Age of farmers influenced cholinesterase inhibition among exposed farmers. AChE levels were lower in younger farmers less than 20 years (23.08 ± 2.84 U/g Hb), and much older farmers aged 60 years and above (25.20 ± 2.34 U/g Hb) compared with the middle-aged farmers of 30–39 years and 40–49 years (29.88 ± 3.58 U/g Hb and 28.63 ± 6.34 U/g Hb) respectively (Table 7). The association between age and AChE inhibition was statistically significant (*p* = 0.046), suggesting the vulnerability of younger and older ages on increased pesticide exposure risks. The analysis of variance (ANOVA) test showed that farmers' exposure to pesticides did not significantly differ among farmers with different working experience and handling of pesticides (*p* = 0.737). However, exposed farmers with working experience of 1–4 years in spraying and handling of pesticides had slightly lower AChE (28.82 ± 2.97 U/g Hb) compared with those worked between 5 and 9 years (30.69 ± 3.23 U/g Hb) and above ten years (30.47 ± 5.07 U/g Hb), but the difference was not statistically significant.

Logistic regression analysis results presented in Table 8 show that an increase in age (10 years interval) increases risks of lower AChE by 6.7% (odds ratio (OR) = 1.067; 95% CI: 0.864; 1.319) while the 1% decrease in BMI increases the probability of risk of having low AChE levels by 86.7% (OR = 0.867; 95% CI: 0.502; 1.496). The decrease in average farm area sprayed per day decreases the probability of farmers having lower AChE levels (OR = 0.001; 95% CI: 0.000; 0.372). Farmers with long working hours have the probability of about three times of having lower AChE levels (OR = 3.497; 95% CI: 1.080; 11.322).

Risk behaviors analysis showed that farmers were aware of and avoided exposure risks behaviors. Eating, smoking, and drinking while spraying were not common behaviors among farmers. Only 22.6% and 27.4% of the farmers ate and drank during pesticides spraying, respectively. Likewise, smoking was not prevalent; 96.4% did not smoke during pesticides spraying (Table 9). There were no statistical differences in the cholinesterase inhibition levels among farmers who ate (*p*=0.171) and drank (*p*=0.069) and those who smoked during spraying (*p*=0.156).

A total of 38 clinical manifestations, which are typical symptoms for organophosphates and carbamates exposure, were reported by farmers. Exposed farmers reported more exposure symptoms than the control group (14.10 ± 7.70 against 6.48 ± 6.62 respectively). Additionally, 40.9% of exposed farmers reported 10–19 exposure symptoms as opposed to 27.6% from the control group. On the contrary, none of the exposed farmers reported no exposure symptom while 24.1% of individuals from the control reported no exposure symptoms. More than a quarter (27.3%) of exposed farmers reported 20 and above exposure symptoms of pesticides exposure (Table 10). There was no significant difference in the number of exposure symptoms between farmers who partially covered and those totally uncovered during pesticides handling (*p*=0.217).

The most reported exposure symptoms which differed significantly between the exposed and control group include tiredness (71.6% versus 15.5%), fatigue (64.8% versus 27.6%), soreness in joints (59.1% versus 20.7%), thirst (56.8% versus 12.1%), headache and weakness (52.3% versus 34.5%), skin irritation (51.1% versus 17.2%), salivation (50.0% versus 8.6%), and abdominal pain (50.0% versus 31.0%) as shown in Table 11. A comparative analysis between the exposed and control indicated a statistically significant difference in the number of exposure symptoms reported (*p* < 0.001). Symptoms for which there was no expected association with exposure to cholinesterase inhibitors were not found more commonly in the exposed farmers. These included loss of appetite, lacrimation, loss of consciousness, and vomiting being not directly linked to exposure symptoms. Only those which significantly differed between the two groups were attributed to pesticides exposure.

Furthermore, the results show statistically significant association (*p* < 0.001) between the number of exposure symptoms and the level of AChE inhibition. This indicates that farmers with more symptoms are more likely to have lower levels of AChE (Table 12).

## 5. Discussion

This study found that organophosphates and carbamate pesticides constitute the main pesticides used. But these pesticides are reported to be responsible for the bulk of acute poisoning cases [32, 33]. Similar pesticides have been previously reported in Tanzania [21, 34], Brazil [35], Australia [17], and Nepal [10]. Both organophosphate and carbamate pesticides are cholinesterase-inhibiting chemicals that induce neurotoxic effects. Previous studies have reported that occupational exposure to a mixture of OPs and CA results in decreased acetylcholinesterase (AChE) activity [9]. The low levels of AChE revealed among the exposed farmers indicate that there was exposure to OPs, carbamates, or a mixture of both OPs and carbamates.

Lower AChE levels in exposed farmers are significantly associated with DNA damage, reactive oxygen stress (ROS), and increased micronuclei frequencies [5, 10, 36–38], indicating that exposed farmers with significantly lower AChE are at risk of genotoxic effects of pesticides exposure. Similar findings had been reported previously [9, 28, 32, 39], but the levels observed in this study are slightly smaller than AChE inhibition reported among Indonesian farmworkers [40]. On the contrary, findings from Iowa and North Carolina, as well as Australia, showed no statistical difference in the AChE levels between the exposed and unexposed individuals [17, 41]. This may be explained by the fact that most farmers in developing countries rely on the use of pesticides that are relatively cheaper but highly toxic while the use of pesticides is highly regulated and monitored in developed countries.

A difference in the level of exposure between men and women was noted. Women farmers, mainly involved in weeding and harvesting of horticultural crops, were more exposed than men in this study, supporting women vulnerability to OPs and CA exposure. These findings support previous studies from Thailand, India, and Indonesia [14, 42, 43]. Women are more exposed than pesticide applicators, possibly because safety training and the use of PPE are usually lower, and the duration of exposure may be higher than that of the applicators. Likewise, women are thought to do less risky work; hence, they get less protection from pesticides exposure [25, 44].

Susceptibility to pesticides exposure is also enhanced by their larger relative fat mass; thus, larger distribution volume for lipophilic organophosphates, which are exceptionally prone to storage in fat tissue [45]. The alarming cholinesterase inhibition among women compared with men justifies further studies on their genetic damage from possible genotoxicity of pesticide exposure. Further studies are also required to investigate pesticide effects on women fertility levels and coexposure of women and their children as women tend to work while their babies are close.

A trend in nutrition status against exposure levels was observed. Farmers with low BMI (underweight) and higher BMIs (Overweight and obese) had significantly lower AChE levels. The observed variations in the levels of exposure may be directly associated with impaired body functioning for undernourished and overnourished individuals. A need for further studies on involved mechanisms for these results is further warranted, including genotoxicity analysis among groups with different nutrition status.

Eating and smoking increase the risks of oral exposure. These risk behaviors, including drinking during pesticides handling, were not a common practice for the farmers. Only 22.6% of the farmers reported eating during pesticides spraying. This is much lower than the proportion of farmers (60%) reported in a previous study from Benin [5]. Lack of significant difference in the level of exposure between farmers who reported eating and smoking and those who did not, therefore, show that the main route of exposure was through dermal exposure. Farmers avoiding eating and smoking during spraying indicate their awareness on the risk behaviors.

From the demographic parameters investigated, age influenced AChE levels. The variation was observed between younger and older farmers with younger and older farmers significantly having lower AChE levels compared with middle-aged farmers. This is contrary to the reported study in Benin [5] where the age of the farmers did not significantly influence the level of AChE. Enzymes involved in the metabolism of pesticides may be highly susceptible to inhibition to the immature immunity (the youth) and compromised immunity (older adults), suggesting that body immunity may be a predisposing factor for vulnerability and susceptibility to pesticides exposure.

Farmers' exposure to pesticides did not differ significantly with the working period of farmers in handling pesticides. These findings are not in support of a previous study in Pakistan [46] that reported strong relationship with duration of work in agriculture and chlorpyrifos and endosulfan levels. The observed similarities in exposure levels among farmers with different working experience with pesticides may be attributed to poor pesticides use practices. Both experienced and unexperienced farmers are equally exposed to pesticide-deriving similar health effects. Likewise, exposure patterns may have changed as its not known whether they used Cholinesterase inhibitors consistently.

Increasing the area sprayed was found to increase the probability of lower AChE levels. As expected, farmers with long working hours have high exposure risks. Increasing operation time results in increased pesticides contact hours, which ultimately fosters the rate of dermal exposure from wet cloths and leaking spraying equipment, which were observed in the field. Likewise, nonstatistically significant association was observed whereby exposure risks were relatively high among farmers who sprayed without break because the time required to liberate the enzyme (AChE) from inhibition is more than the time necessary for the synthesis of a new enzyme, which is more than 30 days after exposure [46]. Farmers breaking more than a month have more time for the enzyme recovery and metabolic detoxification of OPs. Breaking for a reasonable period before embarking on intensive spraying season can reduce the level of exposure among farmers.

Farmers' pesticides handling practices were observed to increase risks of pesticides exposure. The health effects of pesticides exposure observed in this current study may be partly attributed to poor personal protective equipment (PPE). The use of PPE is an essential aspect of personal protection against exposure because most organophosphates are highly lipid-soluble agents and are well absorbed from the skin [25]. The results from this study show that the use of PPE did not significantly reduce pesticides exposure. Insignificant difference between the partially and unprotected farmers suggests that PPE is not effectively used because effective use of PPE had been reported to reduce health hazards from pesticides [6, 47, 48]. Poor use and quality of the PPE used may also account for their insignificant contribution in reducing exposure among farmers.

The general population is environmentally exposed to pesticides due to the modest AChE inhibition in the controls. Similar findings had been previously reported in Pakistan, where non-farmers were found to be exposed [49]. Dietary exposure is assumed to be the main route of exposure. Consumption of pesticides-contaminated crops and the availability of pesticides in the environments resulting from poor disposal of obsolete and empty pesticides containers may account for this exposure, which warrants a critical assessment of safety of horticultural crops locally produced and consumed in Tanzania.

More exposure symptoms were reported among the exposed compared with the control group similar to previous results reported in Nepal, Vietnam, and Indonesia [10, 43]. However, the number reported in this study is far above the average number of symptoms in literature reviewed [6, 50]. High prevalence of tiredness, fatigue, soreness in joints, thirst, skin irritation, salivation and abdominal pain, muscle weakness, and memory loss indicate chronic and neurotoxic effects associated with deficits in neurobehavioral performance and abnormalities in nerve functioning [51]. The statistically significant association between the number of symptoms and the level of AChE, and the fact that our “dummy” symptoms were not elevated; this confirms that the observed symptoms emanated from occupational exposure to pesticides. Consequently, the statistically significant difference in the number of symptoms between exposed and control groups further shows that farmers are occupationally exposed to pesticides in the study areas, raising a public health concern for the farming community.

## 6. Conclusion

Of recent, there had been no biomonitoring exposure evaluation of this kind done by comparing the exposed and unexposed in Tanzania. This study provides evidence of exposure to pesticides in uncontrolled smallholder horticultural production systems. The decreased levels of AChE and positive association with a number of exposure symptoms in exposed farmers signify that the health effects of pesticides exposure are real among the farming population in Tanzania. Women, young (less than 20 years) and older farmers (60 years and above), those with lower BMI, and obese were all at increased risk of pesticide exposure. Decrease in AChE levels among the control group indicates that the general population is at risk of pesticides environmental exposure due to the presence of pesticides in the environment, food chain, and water.

Pesticides use therefore needs to be controlled both for workers and for nonworking populations exposed through other routes of environmental contamination by developing pesticides monitoring and surveillance systems. Capacity building for women workers, in particular, on self-protection and observance of pre-entre and re-entre periods, should be emphasized in the farming communities. Policy on regular monitoring of pesticides exposure is vital. Inclusion of pesticides exposure in national health and epidemiological surveys to consider pesticides exposure as a public health concern among the farming population will raise public awareness of pesticides exposure.

## Figures and Tables

**Table 1:** Demographic characteristics of the study population.

Variable	*n*	%
Region of respondent		
Iringa (Kilolo)	108	71.5
Arusha (Ngarenanyuki)	43	28.5
Category of respondent		
Exposed	90	59.6
Unexposed (control)	61	40.4
Age category of respondent		
30–39 years	43	28.5
20–29 years	35	23.2
40–49 years	32	21.2
50–59 years	26	17.2
60 years and above	9	6.0
Less than 20 years	6	4.0
Sex of respondent		
Male	111	73.5
Female	40	26.5
Working experience with pesticides		
10 years and above	37	53.6
5–9 years	19	27.5
1–4 years	12	17.4
Less than one year	1	1.4
Total	69	100.0

**Table 2:** Chemical families of pesticides used in horticultural production.

Variable	*n*	%
Chemical families		
Organophosphorus	285	97.6
Carbamate	158	54.1
Substituted benzene	101	34.6
Pyrethroid + organophosphorus	84	28.8
Avermectin	82	28.1
Carbamate + acylalanine	67	22.9
Dithiocarbamate	57	19.5
Inorganic fungicide	39	13.4
Pyrethroid	26	8.9
Organochlorine	26	8.9
Pyrethroid + nitroimidazole	25	8.6
Oxadiazines	12	4.1
Conazole	6	2.1
Propionic acid	5	1.7

**Table 3:** Farmers' use of personal protection equipment (PPE).

Variable	*n*	%
Wear gloves when spraying pesticides		
No	78	92.9
Yes	6	7.1
Total	84	100.0
Wear boots when spraying pesticides		
Yes	70	83.3
No	14	16.7
Total	84	100.0
Wear a respirator when spraying pesticides	
No	80	95.2
Yes	4	4.8
Total	84	100.0
Wear a mask when spraying pesticides	
No	76	90.5
Yes	8	9.5
Total	84	100.0
Wear goggles when spraying pesticides	
No	82	97.6
Yes	2	2.4
Total	84	100.0
Wear head covers when spraying pesticides	
No	78	92.9
Yes	6	7.1
Total	84	100.0
Wear overall when spraying pesticides	
No	12	92.3
Yes	1	7.7
Total	13	100.0

**Table 4:** Categories of pesticides poisoning.

Category of pesticide poisoning	Category of respondent				Total		*p* value
	Exposed		Unexposed				
	*n*	%	*n*	%	*n*	%	
24.5–31.3 U/g Hb	61	67.8	24	39.3	85	56.3	*p* < 0.001
≥31.4 U/g Hb	15	16.7	37	60.7	52	34.4	
<24.5 U/g Hb	14	15.6			14	9.3	
Total	90	100.0	61	100.0	151	100.0	

**Table 5:** Comparative levels of AChE inhibition.

Variable	Category of respondent							
	Exposed				Unexposed (control)			
	Sex of respondent				Sex of respondent			
	Male		Female		Male		Female	
	Mean	SD	Mean	SD	Mean	SD	Mean	SD
Cholinesterase inhibition (U/g Hb)	28.38	3.49	26.86	4.95	32.76	4.48	33.08	4.21

**Table 6:** Relationship between BMI and cholinesterase inhibition of exposed farmers.

Variable	WHO BMI classification								Total	
	Underweight (<18.5)		Normal (18.50–24.99)		Overweight (25–29.99)		Obese (>30)			
	Mean	SD	Mean	SD	Mean	SD	Mean	SD	Mean	SD
Cholinesterase inhibition (U/g Hb)	26.73	5.56	28.37	3.32	27.32	4.95	21.90	—	27.97	3.89

**Table 7:** Relationship between age categories and cholinesterase inhibition.

Variable	Age category of respondent												
	Less than 20 years		20–29 years		30–39 years		40–49 years		50–59 years		60 years and above		*p* value
	Mean	SD	Mean	SD	Mean	SD	Mean	SD	Mean	SD	Mean	SD
Cholinesterase inhibition (U/g Hb)	23.08	2.84	27.74	2.85	29.88	3.58	28.63	4.58	26.55	4.00	25.20	2.43	0.046

**Table 8:** Logistic regression analysis for determinants of the outcome variable (AChE < 24.5 Ug/Hb).

Independent variables	*B*	*Z*-values	Sig.	Odd ratios	95.0% CI	
					Lower	Upper
Age categories	0.065	0.367	0.045	1.067	0.864	1.319
BMI categories	−0.143	0.262	0.008	0.867	0.502	1.496
1–4 yrs working with pesticides	−1.369	0.292	0.589	0.254	0.002	36.495
5-yrs working with pesticides	−1.614	0.570	0.450	0.199	0.003	13.147
Average area spread/day	−6.620	5.309	0.021	0.001	0.000	0.372
Working hours/day	1.252	4.363	0.037	3.497	1.080	11.322
Break less than a month before next intensive spray period	−0.763	0.126	0.723	0.466	0.007	31.497
Break for 1-2 months before next intensive spray period	−2.580	0.979	0.322	0.076	0.000	12.551
Constant	−2.792	0.044	0.835	0.061		

**Table 9:** Pesticides exposure risk practices.

Variable	*n*	%
Eat while dealing with pesticides		
No	65	77.4
Yes	19	22.6
Total	84	100.0
Drinking while dealing with pesticides		
No	61	72.6
Yes	23	27.4
Total	84	100.0
Smoking while dealing with pesticides		
No	81	96.4
Yes	3	3.6
Total	84	100.0

**Table 10:** Comparative categories of reported signs and symptoms.

Variable	Category of respondent				Total	
	Exposed		Control		*n*	%
	*n*	%	*n*	%		
Categories of number of symptoms						
1–9 symptoms	28	31.8	25	43.1	53	36.3
10–19 symptoms	36	40.9	16	27.6	52	35.6
20 symptoms and above	24	27.3	3	5.2	27	18.5
No exposure symptoms			14	24.1	14	9.6
Total	88	100.0	58	100.0	146	100.0

**Table 11:** Self-reported exposure signs and reported symptoms.

Variable	Category of respondent			
	Treatment		Control	
	*n*	%	*n*	%
Tiredness	63	71.6	9	15.5
Fatigue	57	64.8	16	27.6
Soreness in joints	52	59.1	12	20.7
Thirst	50	56.8	7	12.1
Headache	46	52.3	20	34.5
Weakness	46	52.3	11	19.0
Skin irritation	45	51.1	10	17.2
Salivation	44	50.0	5	8.6
Abdominal pain	44	50.0	18	31.0
Muscle weakness	42	47.7	14	24.1
Memory loss	42	47.7	17	29.3
Excessive sweating	40	45.5	9	15.5
Blurred vision	40	45.5	18	31.0
Blurred vision associated with excessive tearing	38	43.2	15	25.9
Eye irritation	37	42.0	8	13.8
Nervousness	35	39.8	15	25.9
Moodiness	34	38.6	14	24.1
Perspiration	34	38.6	12	20.7
Irritation of the nose and throat	33	37.5	6	10.3
Productive cough	33	37.5	11	19.0
Drooling	31	35.2	12	20.7
Chest pain	31	35.2	5	8.6
Dizziness	30	34.1	14	24.1
Loss of appetite	28	31.8	22	37.9
Muscle twitches	28	31.8	8	13.8
Red eyes	28	31.8	11	19.0
Nausea	27	30.7	7	12.1
Restlessness	26	29.5	13	22.4
Shortness of breath	24	27.3	6	10.3
Skin rash	21	23.9	3	5.2
Tremor	20	22.7	3	5.2
Lacrimation	20	22.7	13	22.4
Loss of weight	18	20.5	6	10.3
Diarrhea	14	15.9	4	6.9
Loss of consciousness	9	10.2	5	8.6
Vomiting	7	8.0	4	6.9
Confusion	5	5.7	3	5.2
Convulsions	3	3.4		

**Table 12:** Chi-square test for the association between number of exposure symptoms and AChE inhibition.

	Value	df	*p* value
Pearson chi-square	438.000	93	*p* < 0.001
Continuity correction			
Likelihood ratio	371.564	93	*p* < 0.001
Linear-by-linear association	127.415	1	*p* < 0.001
No. of valid cases	146		

## Data Availability

The legal requirements of the research permit as per the National Institute of Medical Research (NIMR) restrict public sharing and access of raw data for ethical concerns. However, authors requiring data may contact the corresponding author for assistance.
